# A Nationwide Survey of Psychological Distress among Italian People during the COVID-19 Pandemic: Immediate Psychological Responses and Associated Factors

**DOI:** 10.3390/ijerph17093165

**Published:** 2020-05-02

**Authors:** Cristina Mazza, Eleonora Ricci, Silvia Biondi, Marco Colasanti, Stefano Ferracuti, Christian Napoli, Paolo Roma

**Affiliations:** 1 Department of Neuroscience, Imaging and Clinical Sciences, University “G.d’Annunzio”, 61100 Chieti-Pescara, Italy; 2 Department of Human Neuroscience, Sapienza University of Rome, 00185 Rome, Italy; eleonoraricci25@gmail.com (E.R.); silviabiondi14@gmail.com (S.B.); marco.colasanti@hotmail.com (M.C.); stefano.ferracuti@uniroma1.it (S.F.); paolo.roma@uniroma1.it (P.R.); 3 Department of Medical Surgical Science and Translational Medicine, Sapienza University of Rome, 00189 Rome, Italy; christian.napoli@uniroma1.it

**Keywords:** COVID-19, pandemic, quarantine, depression, anxiety, stress, mental health intervention

## Abstract

The uncontrolled spread of the coronavirus disease 2019 (COVID-19) has called for unprecedented measures, to the extent that the Italian government has imposed a quarantine on the entire country. Quarantine has a huge impact and can cause considerable psychological strain. The present study aims to establish the prevalence of psychiatric symptoms and identify risk and protective factors for psychological distress in the general population. An online survey was administered from 18–22 March 2020 to 2766 participants. Multivariate ordinal logistic regression models were constructed to examine the associations between sociodemographic variables; personality traits; depression, anxiety, and stress. Female gender, negative affect, and detachment were associated with higher levels of depression, anxiety, and stress. Having an acquaintance infected was associated with increased levels of both depression and stress, whereas a history of stressful situations and medical problems was associated with higher levels of depression and anxiety. Finally, those with a family member infected and young person who had to work outside their domicile presented higher levels of anxiety and stress, respectively. This epidemiological picture is an important benchmark for identifying persons at greater risk of suffering from psychological distress and the results are useful for tailoring psychological interventions targeting the post-traumatic nature of the distress.

## 1. Introduction

The coronavirus disease 2019 (COVID-19) pandemic emerged in Wuhan, China, in late 2019; by the start of 2020, it had spread worldwide. At present, Italy is one of the most affected countries: official data (http://opendatadpc.maps.arcgis.com/apps/opsdashboard/index.html#/b0c68bce2cce478eaac82fe38d4138b1) show that, at the start of the present study (19 March 2020), there were 33,190 confirmed COVID-19 cases, including 3405 deaths and 4440 recoveries. The exceptional spread of COVID-19 in Italy led the government to implement unprecedented quarantine measures, sealing off the northern regions, initially, followed by the entire country.

A recent review of the literature on the psychological effects of quarantine during past epidemics and pandemics (e.g., SARS, H1N1, Ebola, MERS, equine influenza) highlighted that, when comparing the psychological outcomes of quarantined versus non-quarantined persons, the former are more likely to show psychological distress [1]. Furthermore, quantitative studies have underlined that quarantined persons have a high prevalence of psychological symptomatology, including post-traumatic and depressive symptoms, stress, and anxiety [2,3,4,5]. A recent study on the impact of the COVID-19 emergency indicated that “53.8% of respondents rated the psychological impact of outbreak as moderate or severe; 16.5% of respondents reported moderate to severe depressive symptoms; 28.8% of respondents reported moderate to severe anxiety symptoms; and 8.1% reported moderate to severe stress levels” [6] (p. 21). Similarly, Qiu et al. reported that almost 35% of the 52,730 participants in their study showed psychological distress [7].

The international literature has examined—for both general and specific (e.g., health worker) populations—the impact of sociodemographic factors on individuals’ responses to quarantine, levels of distress during health-related emergencies, and other variables associated with a higher likelihood of developing psychological problems. Recent studies [6,7,8], addressing the impact of COVID-19 in China, have suggested that gender was a consistent predictor of psychological outcome: females were more significantly affected by psychological distress than their male counterparts, showing moderate anxiety levels. Regarding age, both young adults (aged 18–30 years) and the elderly (older than 60 years) have been found to exhibit the highest levels of psychological distress, although results have varied across studies. A medical history of chronic illness has also been associated with higher levels of distress [6]. Other sociodemographic variables, such as education level, have yielded mixed results, with both higher and lower education levels associated with higher levels of distress [6,7]. Furthermore, many studies have highlighted significant differences in individuals’ reactions to threat, according to specific personality traits [9,10]. For instance, the literature indicates a relationship between negative affect and detachment—two maladaptive extremes of the five-factor model of personality [11]—and internalizing psychopathology (e.g., depression and anxiety) [12,13,14].

Overall, the aforementioned literature supports the view that imposed isolation has a huge impact on many aspects of people’s lives, causing considerable psychological strain and triggering a variety of psychological problems. Therefore, in the current context of Italy, the negative effects of the quarantine must be addressed alongside the virus outbreak itself. The present study was the first to administer and analyse a nationwide large-scale survey of psychological distress in the general population of Italy during the COVID-19 pandemic. A self-report questionnaire, integrated with the Italian version of the Depression, Anxiety and Stress Scale–21 items (DASS-21) [15], and two scales (Negative Affect and Detachment) of the Personality Inventory for the Diagnostic and Statistical Manual of mental disorders, DSM-5–Brief Form (PID-5-BF) [11], was designed to measure psychological distress during the pandemic, aimed at establishing the prevalence of psychiatric symptoms and identifying risk and protective factors for psychological distress. As measured by the DASS-21, depression assesses dysphoria, anhedonia, lack of incentive, and low self-esteem; anxiety refers to somatic and subjective symptoms of anxiety and an acute response of fear; stress evaluates irritability, impatience, tension, and persistent arousal. As measured by the PID-5-BF, negative affect involves tendencies to experience unpleasant feelings such as anger or anxiety and labile emotionality; detachment involves depressive affect and interpersonal withdrawal.

The results may assist government agencies and healthcare professionals in safeguarding the psychological wellbeing of the community in the wake of the COVID-19 outbreak in Italy and other affected countries.

## 2. Material and Methods

### 2.1. Procedures

We assessed the public’s immediate psychological response to the COVID-19 epidemic in Italy using an anonymous online questionnaire that had been assembled by the research team, inspired by the literature. Data were collected over 5 days (18–22 March 2020). The questionnaire was administered cross-sectionally on an online survey platform, which participants accessed via a designated link. The link was disseminated through the main means of communication and social networks, in order to reach a large number of subjects. Expedited ethics approval was obtained from the Institutional Board of the Department of Human Neuroscience, Faculty of Medicine and Dentistry, “Sapienza” University of Rome (IRB-2020-6), in conformity with the principles embodied in the Declaration of Helsinki.

### 2.2. Setting and Participants

A total of 2812 respondents participated in the survey. Inclusion criteria were: (a) 18 years and older, and (b) living in Italy. The online survey was closed on the sixth day following dissemination of the link. All participants voluntarily responded to the anonymous survey and indicated their informed consent within the survey. The procedures were clearly explained, and participants could interrupt or quit the survey at any point without explaining their reasons for doing so. Two respondents were excluded from the sample because they were younger than 18 years of age, and 44 participants were excluded because they were outside Italy during the outbreak.

The final sample consisted of 2766 participants: 1982 (71.7%) females and 784 (28.3%) males. The mean age of the sample was 32.94 (13.2; range 18–90 years) and most of the sample (*n* = 1194, 43.2%) held a high school diploma and were unmarried (*n* = 1866, 67.5%), students (*n* = 1052, 38.0%), and childless (*n* = 2130, 77%). Furthermore, most participants reported following the advice of the government to stay at home (*n* = 2365, 85.6%). More descriptive statistics, including all characteristics considered, are presented in Table 1.

### 2.3. Present Survey Development

The online questionnaire, which comprised part of a wider research project, covered several areas: (a) sociodemographic data; (b) working life (e.g., whether participants were staying home or going to work); (c) acquaintances infected with COVID-19; (d) loved ones infected with COVID-19; (e) previous physical diseases (e.g., cardiovascular or oncological pathologies); (d) previous stressful situations (e.g., dismissal, mourning); and (h) the psychological impact of COVID-19 on depression, anxiety, and stress levels over the past 7 days.

#### 2.3.1. Sociodemographic Data

Sociodemographic data were collected on biological sex, age, education, marital and parental status, employment status, residential location during the COVID-19 outbreak, and history of stressful situations and medical problems. Moreover, further information related to COVID-19 was collected. Specifically, participants were asked if they were following the government advice to stay at home or if they were continuing to go to work; they were also asked if any acquaintances or loved ones were (or had been) infected with COVID-19.

#### 2.3.2. Psychological Impact and Mental Health

Mental health was measured using the Depression, Anxiety and Stress Scale–21 items (DASS-21) [15].The DASS has been demonstrated to be a reliable and valid measure for assessing mental health in the Chinese population [6,16,17], and it has been applied in studies related to the SARS outbreak [18]. The DASS-21 is a set of three self-report scales designed to measure the emotional states of depression, anxiety, and stress. Each of the three scales contains seven items, divided into subscales with similar content. Items 3, 5, 10, 13, 16, 17, and 21 comprise the Depression subscale; items 2, 4, 7, 9, 15, 19, and 20 comprise the Anxiety subscale; and items 1, 6, 8, 11, 12, 14, and 18 comprise the Stress subscale. All subscales are rated on a four-point Likert scale ranging from 0 (*never*) to 3 (*almost always*). DASS-21 outcome scores are classified into three ranges: average, high, and extremely high. The DASS-21 obtained high reliabilities in the Italian validation study, with Cronbach’s alphas of 0.74, 0.82, and 0.85 for the Anxiety, Depression, and Stress subscales, respectively; Cronbach’s alpha for the total scales was 0.90. In the Lovibond and Lovibond version of the DASS-21 [19], the subscales are scored as follows: normal (0–9), mild (10–12), moderate (13–20), severe (21–27), and extremely severe (28–42) for Depression; normal (0–6), mild (7–9), moderate (10–14), severe (15–19), and extremely severe (20–42) for Anxiety; and normal (0–10), mild (11–18), moderate (19–26), severe (27–34), and extremely severe (35–42) for Stress.

#### 2.3.3. Personality Dysfunction

Personality functioning was investigated using the Personality Inventory for DSM-5–Brief Form–Adult (PID-5-BF) [11]. The PID-5-BF is a 25-item self-rated personality trait assessment scale. It assesses five personality trait domains: negative affect, detachment, antagonism, disinhibition, and psychoticism. Each domain is measured through five items that are rated on a four-point Likert scale ranging from 0 (*very false or often false*) to 3 (*very true or often true*). The overall measure generates scores in the range of 0–75, with higher scores indicating greater overall personality dysfunction. Each trait domain receives a score in the range of 0–5, with higher scores indicating greater dysfunction in that specific personality trait domain. In the present study, we evaluated the dimensions of negative affect and detachment. We selected these two dimensions because the literature indicates a relationship between these traits and internalizing psychopathology (e.g., depression and anxiety) [12,13,14]. The test showed good internal consistency, with Cronbach’s alpha of 0.83.

Descriptive statistics of the DASS-21 and PID-5-BF subscales are reported in Table 2.

## 3. Statistical Analysis

Referring to the community sample descriptive statistics reported by Bottesi et al. [15], the DASS-21 outcome scores for depression, anxiety, and stress were classified into three ranges: average, high, and extremely high. In more detail, cut-offs were set at one and two standard deviations away from the mean, in order to establish average and high levels, respectively. Multivariate ordinal logistic regression models were constructed to capture the associations between sociodemographic variables and DASS-21 depression, anxiety, and stress levels. Further multivariate ordinal logistic regression models were run to compute the effects of personality traits (i.e., negative affect and detachment) on depression, anxiety, and stress levels, together with the sociodemographic variables that resulted as significant in the previous models. Statistical analysis was performed using SPSS Statistics 25.0 (IBM SPSS Statistics, New York, NY). Level of statistical significance was set at *p* < 0.05.

## 4. Results

Associations between sociodemographic variables and DASS-21 depression, anxiety, and stress levels during the COVID-19 outbreak, are presented in Table 3. A multiple ordinal logistic regression analysis was performed with depression, anxiety, and stress levels as the dependent variables; age and education level as covariates; and the other sociodemographic variables as factors.

A second multiple ordinal logistic regression analysis was performed, with depression, anxiety, and stress levels as the dependent variables. Negative affect and detachment were entered as covariates together with the sociodemographic variables that resulted as significant in the previous models, considered or covariates (i.e., age and education level) or factors.

### 4.1. Depression

In relation to depression, 67.3% (*n* = 1859) of respondents had an average level, 17% (*n* = 470) were in the high range, and 434 (15.4%) were in the extremely high range. The prediction model for depression showed goodness of fit to our observed data (χ^2^ (22) = 202.781, *p* < 0.001). The test of parallel lines was not significant (χ^2^ (22) = 16.783, *p* = 0.775). Nagelkerke’s pseudo R^2^ of 0.086 indicated that the significant sociodemographic variables explained approximately 9% of the variability. Lower education levels, female gender, unemployment compared to employment, not having a child, having an acquaintance infected with COVID-19, and having a history of stressful situations and medical problems were associated with higher depression levels.

Again, the second prediction model for depression showed goodness of fit to our observed data (χ^2^ (12) = 721.328, *p* < 0.001). The test of parallel lines was not significant (χ^2^ (12) = 20.953, *p* = 0.051). Nagelkerke’s pseudo R^2^ of 0.280 indicated that the significant sociodemographic variables explained approximately 30% of the variability. Higher levels of negative affect and detachment were significantly associated with higher levels of depression. Education level and working position were excluded from the final model (see Table 4).

### 4.2. Anxiety

In relation to anxiety, 81.3% (*n* = 2247) of respondents had an average level, 7.2% (*n* = 198) were in the high range, and 318 (11.5%) were in the extremely high range. The prediction model for anxiety showed goodness of fit to our observed data (χ^2^ (22) = 166.487, *p* < 0.001). The test of parallel lines was not significant (χ^2^ (22) = 19.765, *p* = 0.598). Nagelkerke’s pseudo R^2^ of 0.083 indicated that the significant sociodemographic variables explained approximately 8% of the variability. Young age, female gender, having a family member infected with COVID-19, and a history of stressful situations and medical problems were associated with higher anxiety levels.

The second prediction model for anxiety also showed goodness of fit to our observed data (χ^2^ (7) = 418.594, *p* < 0.001). The test of parallel lines was not significant (χ^2^ (7) = 3.620, *p* = 0.822). Nagelkerke’s pseudo R^2^ of 0.200 indicated that the significant sociodemographic variables explained 20% of the variability. Higher levels of negative affect and detachment were significantly associated with increased levels of anxiety. Age was excluded from the final model (see Table 5).

### 4.3. Stress

In relation to stress, 2012 (72.8%) of respondents were in the average range, 404 (14.6%) were in the high range, and 347 (12.6%) were in the extremely high range. The prediction model for stress showed goodness of fit to our observed data (χ^2^ (22) = 177.072, *p* < 0.001). The test of parallel lines was not significant (χ^2^ (22) = 23.936, *p* = 0.351). Nagelkerke’s pseudo R^2^ of 0.079 indicated that the significant sociodemographic variables explained approximately 8% of the variability. Higher levels of stress were associated with young age, female gender, having to go out to work, having an acquaintance infected with COVID-19, and a history of stressful situations and medical problems.

The second prediction model for stress also showed goodness of fit to our observed data (χ^2^ (8) = 530.980, *p* < 0.001). The test of parallel lines was not significant (χ^2^ (8) = 13.842, *p* = 0.086). Nagelkerke’s pseudo R^2^ of 0.222 indicated that the significant sociodemographic variables explained 22% of the variability. Higher levels of negative affect and detachment were significantly associated with increased levels of anxiety. A history of stressful episodes and medical problems was excluded from the final model (see Table 6).

## 5. Discussion

A recent review of the literature underlined a high prevalence of psychological symptomatology among quarantined individuals [1], including post-traumatic and depressive symptoms, stress, and anxiety [2,3,4,5]. Thus, the present study sought to survey psychological distress in Italy during the initial phase of the COVID-19 pandemic outbreak, with the aim of establishing the prevalence of psychiatric symptoms and identifying risk and protective factors for psychological distress among sociodemographic and personality variables.

Overall, our results showed an increased percentage of people with high and very high levels of distress compared to the European epidemiological statistics [20,21]. For example, the latest data (https://www.epicentro.iss.it/mentale/epidemiologia-italia) indicate that, in Italy, only about 6% of adults aged 18–69 report depressive symptoms, compared to the higher percentages of high and very high levels of distress found here. In line with the aforementioned review [1], the increased frequency of distress found in the current sample could be interpreted as COVID-19 related, although further studies should confirm this association.

The results indicated that female gender was associated with increased anxiety, depression, and stress. This finding is in line with the results of previous studies [6,7], which have consistently found an association between female gender and increased psychological distress. The finding may also be linked to evidence in the international literature that women tend to be more vulnerable to experiencing stress and developing post-traumatic symptoms [22]. Together with female gender, the personality domains of negative affect and detachment were also found to be associated with high levels of anxiety, depression, and stress in the present research. This result also accords with the literature [12,13,14], which indicates that both of these personality traits are good indices of internalizing psychopathology.

Another finding was the association between a history of stressful situations and increased anxiety and depression. This result adheres to the findings of researches highlighting that persons with a history of trauma suffer from long-term effects of the trauma [23], often in the form of psychological symptoms that can flare up or re-emerge during situations of psychological uncertainty, such as that of the current COVID-19 outbreak.

The present study also found an association between a history of medical issues and increased anxiety and depression. This finding echoes that of previous studies indicating that chronic illness or a self-evaluation of poor health is associated with increased psychological distress [6,16]. A possible explanation for this result is that persons with a history of medical problems who also perceive their health as poor might feel more vulnerable to contracting a new disease [24].

In the present research, only young age was found to be associated with increased stress. The literature reports mixed results for this variable, indicating a greater psychological impact for both young adults and the elderly [7,25]. Some authors have suggested that greater anxiety amongst the younger population may be due to their greater access to information through social media, which can easily trigger stress [26]. Otherwise, we found no association between participants’ age and depression, as also reported by Hawryluck et al. [2], who studied the psychological outcomes of the SARS quarantine.

Our results showed that having an acquaintance infected with COVID-19 was associated with increased depression and stress, whereas having an infected relative was associated with increased anxiety. In contrast, Wang et al. reported that neither having an infected acquaintance nor having an infected relative was associated with psychological distress, although their participants showed high levels of concern about the health of their family members [6].

To the best of our knowledge, the present study represents the first attempt to provide data on the association between having to leave one’s domicile for work and stress levels during the COVID-19 outbreak. In more detail, the results showed that having to leave one’s domicile for work was associated with increased stress. The health-related measures issued by the Italian government indicate that only a limited number of key workers (e.g., pharmacists, supermarket workers, etc.) are permitted to work outside their domiciles. Such workers tend to have a high level of contact with the public, and are thus at greater likelihood of being infected; this may increase their stress levels.

Finally, the present study found that not having children was associated with depression. This result does not adhere to the prior findings [25], which have indicated that having one child is related to more negative psychological outcomes than having no children.

## 6. Conclusions

Overall, female gender, negative affect, and detachment were associated with higher levels of psychological distress. Having an acquaintance infected with COVID-19 increased both depression and stress, whereas a history of stressful situations and medical problems raised depression and anxiety levels. Finally, having a family member infected with COVID-19 and being young in age and needing to leave one’s domicile to go to work were found to increase anxiety and stress levels, respectively.

As the first step, this epidemiological picture could facilitate the identification of persons at greater risk of suffering from psychological distress, which can have psychopathological consequences and impair functioning. However, only 30% (maximum) of the variability in depression, anxiety, and stress was explained by sociodemographic and personality factors. This means that the majority of the increase in distress levels noted in this research was likely related to the maladaptive and traumatic course of the pandemic—the effects of which can only be assessed empirically at the end of the emergency. Thus, while these results provide an important benchmark for identifying subjects at risk, they may also be useful for tailoring psychological interventions targeting the post-traumatic nature of the distress.

One limitation of this study is that, not having a DASS-21 baseline of pre-pandemic data, accurate pre–post analyses could not be conducted; therefore, we cannot be sure of any increase in distress levels or whether any increase (if validated) was COVID-19 related. However, comparing the mean scores obtained for depression, anxiety, and stress between the DASS-21 normative sample and the sample gathered in this study, we observed a difference: scores of our sample were higher than those of the normative sample, for all three dimensions (e.g., for depression, the normative mean was 3.5 (3.2), whereas in our sample it was 5.34 (4.8)).

Our results offer a general picture of the psychological impact of COVID-19 on the Italian population, providing a baseline for future research on the impact of COVID-19 throughout the rest of the pandemic. However, further studies should carry out multilevel analysis (e.g., grouping for age, gender, or geographical location), with the aim of differentiating distress level in order to develop more targeted interventions.

## Figures and Tables

**Table 1:** Descriptive statistics of the sample.

Characteristic	Group	*n* (%)
Gender	Female	1982 (71.6%)
	Male	784 (28.4%)
Age		2766 (100%)
*M* (*SD*)	32.94 (13.2)	
Min–Max	18–90	
Citizenship	Italian	2739 (99%)
	Foreign	27 (1.0%)
Region of Residence	Nord	565 (20.4%)
	Center	1622 (58.7%)
	Sud	576 (20.8%)
Education	Primary school diploma	3 (0.1%)
	Middle school diploma	104 (3.8%)
	High school diploma	1194 (43.2%)
	Graduate	1098 (39.7%)
	Postgraduate	367 (13.3%)
Marital Status	Unmarried	1863 (67.4%)
	Married	759 (27.5%)
	Separated/Divorced	114 (4.1%)
	Widower	27 (1.0%)
Occupation	Employee	1049 (37.9%)
	Freelancer	437 (15.8%)
	Unemployed	209 (7.6%)
	Student	1052 (38.0%)
	Retired	19 (0.7%)
Child(ren) in House	No	2130 (77%)
	Yes	636 (23%)
Moved after the Onset of COVID-19 Emergency	No	2419 (87.5%)
	Yes	120 (4.3%)
	Yes, to get Closer to Loved Ones	227 (8.2%)
Spending Social Distancing Period with	Family	2072 (75.0%)
	Alone	250 (9.0%)
	Roommate(s)	185 (6.7%)
	Partner	212 (7.7%)
	Other(s)	44 (1.6%)
Condition	Must go to work	398 (14.4%)
	Can stay at home	2365 (85.6%)
Quarantine	No	2446 (88.4%)
	Yes, with family	230 (8.3%)
	Yes, alone	90 (3.3%)
Number of Times you Leave Your Domicile Each Day	0–1	2559 (92.5%)
	2	162 (5.9%)
	2+	45 (1.6%)
Reason for Leaving Domicile	Key worker	435 (15.7%)
	Health reasons	78 (2.8%)
	Return home	111 (4.0%)
	State of need	2142 (77.4%)
Use of Media during Social Distancing: Similar Levels to Before (1) Slightly More than Before (2) Much More than Before (3)	Telephone	(1) 794 (28.7%)
		(2) 1113 (40.2%)
		(3) 859 (31.1%)
	WhatsApp	(1) 641 (23.2%)
		(2) 997 (36%)
		(3) 1128 (40.8%)
	Facebook	(1) 1586 (57.3%)
		(2) 680 (24.6%)
		(3) 500 (18.1%)
	Instagram	(1) 1461 (52.8%)
		(2) 732 (26.5%)
		(3) 573 (20.7%)
	Skype	(1) 642 (23.2%)
		(2) 652 (23.6%)
		(3) 1472 (53.2%)
Use of Social Media (Hours)	1–2	981 (35.5%)
	3–5	1173 (42.4%)
	5–8	423 (15.3%)
	8–10	130 (4.7%)
	10+	59 (2.1%)
Infected Acquaintances	No	2156 (78%)
	Yes	607 (22%)
Deaths among Infected Acquaintances	Yes	104 (3.8%)
Infected Loved Ones	No	2605 (94.3%)
	Yes	158 (5.7%)
History of Stressful Situations	No	1698 (61.5%)
	Yes	1065 (38.5%)
History of Medical Problems	No	2057 (74.4%)
	Yes	706 (25.6%)
Psychological Support or Psychotherapy	No	2182 (78.9%)
	Yes	584 (21.1%)
Application of Health-related Measures	From Late February	876 (31.7%)
	From the First Days of March	1206 (43.6%)
	From the Second Week of March	650 (23.5%)
	Not Applied	9 (0.3%)
	Only for a Few Days	25 (0.9%)
Social Support from Government	Quite Enough	1042 (37.7%)
	Very Sufficient	55 (2.0%)
	Not Enough at all	276 (10.0%)
	Not Enough	888 (32.1%)
	Enough	505 (18.3%)
Government Information Reliability	Quite Reliable	1779 (64.3%)
	Very Reliable	554 (20.0%)
	Not Reliable at all	48 (1.7%)
	Unreliable	385 (13.9%)
Detailed Government Information	Quite Detailed	1358 (49.1%)
	Detailed	619 (22.4%)
	Very Detailed	180 (6.5%)
	Not Detailed at all	74 (2.7%)
	Not very Detailed	535 (19.3%)
Frequency of Updates on COVID-19	No	27 (1.0%)
	Yes, Everyday	1401 (50.7%)
	Yes, Sometimes	412 (14.9%)
	Yes, Many Times per Day	926 (33.5%)

**Table 2:** Descriptive Statistics of Depression, Anxiety and Stress Scale–21 items (DASS-21) and Personality Inventory for DSM-5–Brief Form (PID-5-BF) Subscales.

Variables	M (*SD*) Min.–Max.	*n* (%)
DASS-21 Depression Subscale	5.34 (4.81) 0−21	2766 (100%)
Average		1860 (67, 2%)
High		470 (17, 0%)
Very High		436 (15, 8%)
DASS−21 Anxiety Subscale	2.89 (3.69) 0−21	2766 (100%)
Average		2249 (81, 3%)
High		198 (7, 2%)
Very High		319 (11, 5%)
DASS−21 Stress Subscale	7.43 (5.45) 0−21	2766 (100%)
Average		2014 (72, 8%)
High		404 (14, 6%)
Very High		348 (12, 6%)
PID-5-BF Negative Affect	5.55 (3.08) 0−15	2766 (100%)
PID-5-BF Detachment	3.38 (2.78) 0−13	2766 (100%)
PID-5-BF Total	19.58 (10.74) 0−61	2766 (100%)

**Table 3:** Associations between sociodemographic variables and DASS-21 depression, anxiety, and stress levels during the COVID-19 outbreak.

		Depression					Anxiety					Stress				
Predictor	Descriptives	Estimate	EXP(B)	95% C.I.		*p*	Estimate	EXP(B)	95% C.I.		*p*	Estimate	EXP(B)	95% C.I.		*p*
Age	32.94 (13.2)	−0.007	0.993	−0.020	0.005	0.236	**−0.026**	**0.974**	**−0.042**	**−0.011**	**0.001**	−0.024	0.976	−0.037	−0.011	**<0.001**
Education Level	High School Diploma-Graduate	**−0.239**	**0.787**	**−0.355**	**−0.123**	**<0.001**	−0.108	0.898	−0.250	0.034	0.135	−0.077	0.926	−0.200	0.046	0.219
Gender, ref. Male	71.6%	**0.638**	**1.893**	**0.445**	**0.830**	**<0.001**	**1.179**	**3.251**	**0.899**	**1.459**	**<0.001**	0.918	2.504	0.699	1.136	**<0.001**
Working Position, ref. Unemployed																
Employee	37.9%	**−0.330**	**0.719**	**−0.648**	**−0.011**	**0.043**	0.087	1.091	−0.318	0.492	0.674	−0.227	0.797	−0.568	0.114	0.192
Freelancer	15.8%	−0.308	0.735	−0.669	0.052	0.093	0.105	1.111	−0.350	0.559	0.652	−0.263	0.769	−0.649	0.124	0.183
Retired	0.7%	−0.562	0.570	−1.857	0.733	0.395	0.766	2.151	−0.519	2.051	0.243	−0.484	0.616	−2.014	1.045	0.535
Student	38.0%	−0.272	0.762	−0.589	0.044	0.092	0.014	1.014	−0.390	0.418	0.945	−0.249	0.780	−0.586	0.089	0.149
Marital status, ref. Widower																
Unmarried/Single	67.4%	0.083	1.087	−0.837	1.003	0.860	−0.222	0.801	−1.309	0.866	0.690	−0.301	0.740	−1.299	0.697	0.554
Separated/Divorced	4.1%	−0.182	0.833	−1.157	0.793	0.714	−0.346	0.708	−1.526	0.834	0.565	−0.525	0.592	−1.607	0.556	0.341
Married/Cohabiting	27.5%	−0.331	0.718	−1.230	0.568	0.470	−0.096	0.908	−1.157	0.965	0.859	−0.337	0.714	−1.313	0.640	0.499
Having child, ref. Yes	77%	**0.393**	**1.481**	**0.031**	**0.755**	**0.033**	−0.061	0.941	−0.491	0.368	0.779	0.134	1.143	−0.243	0.512	0.485
Region, ref. South																
North	20.4%	−0.051	0.950	−0.305	0.203	0.692	−0.140	0.869	−0.451	0.171	0.378	−0.107	0.899	−0.376	0.162	0.434
Center	58.7%	−0.056	0.946	−0.262	0.151	0.596	−0.090	0.914	−0.341	0.160	0.480	−0.088	0.916	−0.306	0.130	0.427
Living with, ref. Alone																
Family	75.0%	−0.097	0.908	−0.405	0.210	0.534	0.023	1.023	−0.376	0.423	0.909	0.244	1.276	−0.106	0.595	0.172
Roommates	6.7%	0.170	1.185	−0.231	0.572	0.406	0.183	1.200	−0.330	0.695	0.485	0.427	1.533	−0.019	0.873	0.060
Partner	7.7%	−0.105	0.900	−0.525	0.314	0.623	0.032	1.033	−0.494	0.558	0.905	−0.036	0.965	−0.508	0.437	0.883
Other	1.6%	−0.131	0.877	−0.814	0.552	0.707	0.580	1.786	−0.188	1.348	0.139	0.550	1.733	−0.149	1.25	0.123
Going to work, ref. Staying at home	14.4%	0.049	1.050	−0.214	0.313	0.713	−0.002	0.998	−0.327	0.323	0.991	0.380	**1.462**	**0.111**	**0.649**	**0.006**
Acquaintance infected by COVID−19, ref No	22%	**0.293**	**1.340**	**0.084**	**0.502**	**0.006**	0.165	1.179	−0.090	0.420	0.204	0.266	**1.305**	**0.046**	**0.486**	**0.018**
Family member infected by COVID-19, ref No	5.7%	−0.063	0.939	−0.437	0.311	0.741	0.520	**1.682**	**0.108**	**0.932**	**0.013**	0.217	1.242	−0.159	0.593	0.258
History of stressful situations, ref. No	38.5%	**0.275**	**1.317**	**0.109**	**0.441**	**0.001**	0.301	**1.351**	**0.099**	**0.502**	**0.003**	0.190	**1.209**	**0.014**	**0.366**	**0.035**
History of medical problems, ref. No	25.6%	0.387	1.473	0.198	0.576	**<0.001**	0.536	**1.709**	**0.313**	**0.76**	**<0.001**	0.308	**1.361**	**0.106**	**0.509**	**0.003**

*Note. n* = 2763; three missing data were registered. The bold indicates the significance of the result.

**Table 4:** Association between significant sociodemographic variables, personality traits, and DASS-21 depression during the COVID-19 outbreak (*n* = 2766).

Predictor	Estimate	EXP(B)	95% C.I.		*p*
Education level	–0.059	0.943	–0.181	0.063	0.345
Gender, ref. Male	**0.618**	**1.855**	**0.411**	**0.826**	**<0.001**
Working position, ref. Unemployed					
Employee	–0.213	0.808	–0.544	0.119	0.208
Freelancer	–0.157	0.855	–0.535	0.221	0.416
Retired	–0.561	0.571	−1.877	0.755	0.403
Student	–0.167	0.846	–0.496	0.162	0.320
Having Child, ref. Yes	**0.563**	**1.756**	**0.304**	**0.821**	**<0.001**
Acquaintance Infected by COVID−19, ref No	**0.317**	**1.373**	**0.117**	**0.518**	**0.002**
History of Stressful Situations, ref. No	**0.239**	**1.270**	**0.064**	**0.414**	**0.008**
History of Medical Problems, ref. No	**0.202**	**1.224**	**0.003**	**0.402**	**0.047**
Negative Affect	**0.214**	**1.239**	**0.179**	**0.249**	**<0.001**
Detachment	**0.196**	**1.217**	**0.16**	**0.232**	**<0.001**

The bold indicates the significance of the result.

**Table 5:** Association between significant sociodemographic variables, personality traits, and DASS-21 anxiety during the COVID-19 outbreak (*n* = 2766).

Predictor	Estimate	EXP(B)	95% C.I.		*p*
Age	–0.007	0.993	–0.016	0.002	0.107
Gender, ref. Male	**1.123**	**3.074**	**0.834**	**1.412**	**<0.001**
Family Member Infected by COVID-19, ref No	**0.767**	**2.153**	**0.379**	**1.156**	**<0.001**
History of Stressful Situations, ref. No	**0.282**	**1.326**	**0.073**	**0.49**	**0.008**
History of Medical Problems, ref. No	**0.393**	**1.481**	**0.159**	**0.626**	**0.001**
Negative Affect	**0.234**	**1.264**	**0.192**	**0.277**	**<0.001**
Detachment	**0.083**	**1.087**	**0.042**	**0.124**	**<0.001**

The bold indicates the significance of the result.

**Table 6:** Association between significant sociodemographic variables, personality traits, and DASS-21 stress during the COVID-19 outbreak (*n* = 2766).

Predictor	Estimate	EXP(B)	95% C.I.		*p*
Age	**–0.017**	**0.983**	**–0.025**	**–0.009**	**<0.001**
Gender, ref. Male	**0.875**	**2.399**	**0.646**	**1.104**	**<0.001**
Going to Work, ref. Staying at Home	**0.442**	**1.556**	**0.175**	**0.708**	**0.001**
Acquaintances Infected by COVID-19, ref No	**0.347**	**1.415**	**0.141**	**0.552**	**0.001**
History of Medical Problems, ref. No	0.118	1.125	–0.093	0.329	0.274
History of Stressful Situations, ref. No	0.142	1.153	–0.041	0.325	0.128
Negative Affect	**0.233**	**1.262**	**0.196**	**0.27**	**<0.001**
Detachment	**0.104**	**1.110**	**0.067**	**0.14**	**<0.001**

The bold indicates the significance of the result.
